# JAHEE external evaluation

**DOI:** 10.1093/eurpub/ckac129.435

**Published:** 2022-10-25

**Authors:** M Balaj

**Affiliations:** Center for Global Health Inequality Research, Norwegian University of Science and Technology, Oslo, Norway

## Abstract

The general aims and evaluation blocks concerned the extent to which JAHEE achieved its objectives, and the identification of lessons learned for future health inequalities programmes. More specific aims included: • Producing knowledge: to understand the outcomes of JAHEE and build knowledge on health inequalities. • Influencing decision-making: to produce results that can guide the development of policies and initiatives tackling health inequalities. • Accountability: to clarify responsibilities and outcomes of JAHEE. • Lessons learned: to provide recommendations for future initiatives.

The evaluation was grounded in five evaluation blocks: relevance, effectiveness and impact, sustainability, dissemination, and added value. The main results will be presented.

